# Depressão, Doença Cardiovascular e Sexo Feminino: Uma Tríade Subestimada

**DOI:** 10.36660/abc.20220858

**Published:** 2023-06-23

**Authors:** Walkiria Samuel Avila, Maria Alayde Mendonça Rivera, Ivan Romero Rivera

**Affiliations:** 1 Hospital das Clínicas Faculdade de Medicina Universidade de São Paulo São Paulo SP Brasil Instituto do Coração do Hospital das Clínicas da Faculdade de Medicina da Universidade de São Paulo, São Paulo, SP – Brasil; 2 Universidade Federal de Alagoas Faculdade de Medicina Maceió AL Brasil Universidade Federal de Alagoas – Faculdade de Medicina, Maceió, AL – Brasil

**Keywords:** Depressão, Doenças Cardiovasculares, Sexo Feminino, Sistema Imunitário, Eixo Hipotálamo Hipófise Adrenal, Sistema Renina Angiotensina Aldosterona

## Introdução

A depressão é uma doença global que acomete cerca de 330 milhões de pessoas, o que corresponde a 4,4% da população mundial, e a coloca como a segunda causa de incapacitação nos EEUU.^1^ No Brasil, estima-se que 5,8% da população, ou seja, 11 milhões de pessoas, sofram dessa doença, colocando o país como o primeiro na América Latina, e o segundo das Américas em número de casos.^1^ Acrescentam-se os dados divulgados pela Pesquisa Nacional de Saúde (PNS) realizada em 2013 em que se verificou que 78,8% dos brasileiros com sintomas depressivos importantes, não recebiam nenhum tipo de tratamento.^2^

### Depressão, mulheres e risco cardiovascular

As mulheres, a partir da adolescência, apresentam duas vezes maior propensão a desenvolver depressão quando comparadas aos homens e, quanto mais precoce é o início da doença, mais grave é o prognóstico. Estima-se que cerca de 20 a 25% das mulheres sofrerão pelo menos um episódio de depressão ao longo da vida, com elevada taxa de recorrência da doença, que por sua vez, está associada a risco aumentado de doença cardiovascular (DCV).^3^

**Figura 1:**
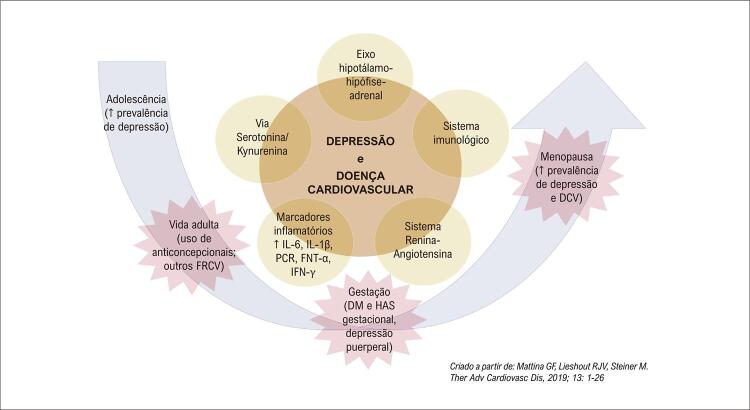
– Fisiopatologia da depressão e doença cardiovascular.

Nesse cenário, o mundo contemporâneo destaca a DCV como a principal causa de morte em ambos os sexos, representando 56% da mortalidade entre as mulheres.^4^ Vale destacar que a depressão é identificada com muita frequência entre pacientes que apresentam DCV, além de haver indícios que depressão e DCV apresentam uma relação bidirecional, o que equivale dizer, que ambas são preditivas “entre si”, e em conjunto deflagram eventos cardíacos mais graves ( Figura 1 ).^5 , 6^

Há evidências de que depressão e DCV compartilham uma patogenia relacionada ao sistema imunológico, com a hipótese de que a persistência de um estado inflamatório crônico, com maior expressão de citocinas inflamatórias, proteínas de fase aguda e moléculas de adesão, quando associado à desregulação do eixo hipotálamo-pituitário-adrenal, do sistema renina-angiotensina-aldosterona e vias serotonina-kynurenina^7^ contribuem para o desenvolvimento de ambas as doenças ( Figura 1 ).^7^

As razões da predominância em mulheres ainda não estão esclarecidas, contudo admite-se que além da predisposição genética e de fatores biológicos, psicossociais e familiares, existe uma conexão da depressão com as flutuações hormonais, moduladas pelos níveis de estrogênio e progesterona, particularmente aquelas inerentes ao ciclo gravídico-puerperal e a menopausa ( Figura 1 ).^8^

### Depressão puerperal

Nesse contexto, vale ressaltar a importância da depressão puerperal, com prevalência no Brasil de cerca de 20,5%, que pode estar subestimada, em razão da falta de diagnóstico e omissão na notificação.^9^ É oportuno mencionar uma recente pesquisa que demonstrou uma proporção maior de infarto do miocárdio e acidente vascular cerebral entre mulheres que sofreram de depressão puerperal, tema que merece futuros estudos no rastreamento de um novo preditivo de risco cardiovascular na mulher.^10^

O quadro clínico da depressão puerperal, apresentado na tabela 1 não difere do clássico da população em geral e pode ter início na gestação ou em até quatro semanas após o parto.^11^ Dentre os sintomas, destacam-se mudanças do humor, perda de interesse pela vida, sentimento de baixa estima e ideação suicida/homicida. Este quadro pode progredir para a psicose (agitação, confusão, delírios), e culminar com o suicídio.

**Tabela 1:** – Características da depressão do gênero feminino e da depressão puerperal

	Depressão	Depressão pós-parto
Fatores de Risco	História familiar de depressão Desequilíbrio em neurotransmissores cerebrais Doenças crônicas (esclerose múltipla, diabetes mellitus, câncer) Dor crônica, alterações do sono Flutuações hormonais (pré-menstrual, gestação, perimenopausa) Alterações do ritmo circadiano Deficiência nutricional Estresse, Perda, luto Medicamentos (anticonvulsivantes, benzodiazepínicos, corticoides) Abuso de álcool e outras drogas	Depressão durante a gestação Multíparas (≥ 3 partos ) Estresse grave durante a gravidez Abuso sexual atual ou anterior Insatisfação conjugal ou do parceiro Complicações referentes ao parto ou ao recém-nascido Sentimentos mistos sobre a gravidez (planejada ou não) Falta de apoio emocional do cônjuge, parceiro, família/amigos **FATORES DE PROTEÇÃO** Maior escolaridade materna e paterna Presença do cônjuge ou companheiro Apoio do pai da criança e da família durante a gravidez
Sintomas observados	Humor deprimido; Perda de interesse ou prazer pelas atividades habituais; Insônia ou hipersonia; Perda ou ganho de peso significativo (> 5% /mês), diminuição ou aumento do apetite Retardo psicomotor ou agitação Fadiga e baixa estima; Diminuição da capacidade de concentração ou tomar decisões Pensamentos de inutilidade ou culpa excessiva ou inadequada Pensamentos recorrentes de morte, ou ideação suicida, ou tentativa de suicídio	Mudanças no apetite e hábitos alimentares Fadiga extrema, Insônia Mudanças de humor (irritabilidade, raiva e tristeza) Falta de interesse em atividades prazerosas ou favoritas Baixa estima ou capacidade de concentração Isolamento social, incluindo o bebê, associado a sentimento de culpa/vergonha Sentimentos de tristeza, inquietação, ansiedade ou desesperança Queixas persistentes (dor de cabeça, estomago ou outras) Pensamentos sobre ferir a si mesmo ou ao seu bebê (ideação suicida/homicida)
Ferramenta Diagnóstica	Questionário de Saúde do Paciente de 2 Perguntas (PHQ-2). Questionário de Saúde do Paciente de 9 Perguntas (PHQ-9).	Escala de Depressão Pós-natal de Edimburgo (EPDS).
Tratamento	Psicoterapia (terapia cognitivo-comportamental; terapia interpessoal; grupos de suporte); Antidepressivos: inibidores seletivos da recaptação de serotonina; inibidores da recaptação da serotonina /norepinefrina (exceto fluoxetina e paroxetina, que inibem o efeito do tamoxifeno, não devendo ser usados em pacientes com câncer de mama); inibidores seletivos da recaptação da norepinefrina/dopamina; antagonistas do receptor alfa-1; moduladores da serotonina. Antidepressivos tricíclicos devem ser evitados em pacientes com doença cardiovascular estrutural pelos seus efeitos na condução do estímulo cardíaco. Exercícios físicos Eletroconvulsoterapia (depressão grave) Orientação para a busca de fatores psicológicos positivos	Psicoterapia (terapia cognitivo-comportamental; terapia interpessoal; grupos de suporte). Antidepressivos: inibidores seletivos da recaptação de serotonina no tratamento inicial (sertralina é a droga preferencial, por sua baixa concentração no leite materno); Outras drogas indicadas: paroxetina, duloxetina, nortriptilina e imipramina; Antidepressivos tricíclicos podem ser considerados o tratamento de primeira escolha, caso haja história de tratamento prévio com sucesso e não existam contraindicações para seu uso, como a probabilidade de suicídio; doxepina é contraindicada (depressão respiratória; sedação; hipotonia) Brexanolona IV em casos graves (disponível nos EUA) Exercícios físicos Outras terapias (yoga; massagens; técnicas de relaxamento; meditação) Eletroconvulsoterapia (depressão grave, com alucinações, ilusões, pensamentos suicidas) A lactação deve ser incentivada pelos benefícios na relação mãe e filho; devem ser discutidos os riscos do tratamento para o bebê e os riscos para a dinâmica familiar de não tratar a depressão.
Prognóstico	O não reconhecimento/tratamento da depressão pode causar: a) impacto negativo no reconhecimento e controle dos fatores de riscos da doença cardiovascular; b) aumento na incidência de eventos agudos	O não reconhecimento/tratamento da depressão puerperal pode causar: a) aumento do risco de suicídio e infanticídio; b) ruptura do vínculo mãe-filho, com danos físicos/emocionais de impacto negativo no crescimento e desenvolvimento da criança (tendência à obesidade; dificuldades nas interações sociais)

A depressão puerperal prejudica a qualidade de vida, fragiliza o vínculo materno-infantil, favorece a discórdia conjugal e motiva o infanticídio, além do suicídio materno. Nesse aspecto, o fato mais alarmante é que a depressão puerperal é uma complicação psiquiátrica que aumenta em cinco vezes a tentativa de suicídio, e foi incluída como a causa entre 30 e 50% das gestantes que se suicidaram.^12^ À vista das razões apresentadas, a perspicácia do profissional médico no diagnóstico da depressão na mulher, é de extrema importância, vez que 10 a 20% das pacientes negam a doença e omitem os sintomas.^13^

### Depressão e perimenopausa

Ainda no ciclo biológico da mulher, a perimenopausa é uma janela de vulnerabilidade para o desenvolvimento da depressão, mesmo em mulheres sem histórico da doença.

Dentre os fatores de risco para a depressão na perimenopausa destacam-se aqueles relacionados à saúde mental, em particular a ansiedade e transtornos depressivos anteriores, e outras condições como a raça negra, obesidade, eventos trágicos no decorrer da vida e isolamento social.^14^ Os “gatilhos” da depressão na perimenopausa encontram-se nas mudanças de carreira ou de relacionamento, na conscientização do envelhecimento, na transformação da aparência física, doença pessoal ou familiar, além da conhecida síndrome do “ninho vazio”.

Os sintomas habituais da depressão são superpostos aos específicos da menopausa, tais como as ondas de calor, suores noturnos, distúrbios do sono e da libido, e alterações cognitivas, que dificultam o diagnóstico da doença. Nesse sentido, a aplicação de questionários convencionais^13^ ( *Menopause Rating Scale* MRS, *Menopause Quality of Life Questionnaire-* MENQOL, *Greene Climacteric Scale, Utian Quality-of-Life Scale* ) pode contribuir na interface de sintomas (menopausa, ansiedade e depressão), e direcionar para a abordagem segura e eficaz da doença.^15^

### Depressão, COVID-19 e mulheres

Uma análise abrangente sobre a interação entre gênero, depressão, DCV e COVID-19, destacou uma prevalência combinada de 25% (95% IC: 18%-33%) de depressão na população geral, resultante dos efeitos da pandemia da COVID-19. Curiosamente, as mulheres parecem ter complicações menos graves em curto prazo, quando comparadas aos homens, porém sofrem de complicações maiores da COVID-19 ao longo do tempo, incluindo a depressão e piora nos hábitos de vida, aumentando o risco cardiovascular.^16^

O sexo feminino tem sido o destaque significativo para a piora do estado de saúde mental e sintomas de depressão, provavelmente em razão da maior prevalência de transtornos depressivos e de ansiedade preexistentes, do estresse ambiental constante, e da violência doméstica, que intensificaram durante a pandemia pela COVID-19. Além do mais, as mulheres vivenciam situações de estresse relacionadas ao período reprodutivo, tais como problemas de fertilidade, gravidez, aborto espontâneo, depressão pós-parto e violência por parceiro íntimo.

Nesse cenário, um estudo multicêntrico que teve o objetivo de avaliar a saúde mental de mulheres no período gravídico-puerperal, durante a pandemia da COVID-19, mostrou uma prevalência de 15% de sintomas depressivos maiores entre 3.907 gestantes e de 13% entre 5.134 lactantes. Esse estudo destaca a importância de monitorar a condição mental perinatal durante pandemias e outras crises sociais, para a salvaguarda da saúde materno-infantil.^17^

### Diagnóstico e tratamento da depressão nas mulheres

A recomendação usual para o diagnóstico da depressão é a aplicação do método conhecido como *Patient Health Questionnaire (PHQ)-2* , que inclui duas questões e, diante da suspeita da doença, utiliza-se o chamado *Patient Health Questionnaire (PHQ)-9* , que contém nove questões, para a confirmação do diagnóstico. A utilização de ambos os métodos de investigação da depressão na prática clínica tem demonstrado boa sensibilidade, especificidade e valor preditivo negativo. Ambos os métodos estão respectivamente disponíveis nos sites https://bit.ly/2VvPHIG (PHQ-2) e https://bit.ly/2PY3INz (PHQ-9).

Para a investigação da depressão puerperal recomenda-se a aplicação da Escala de Depressão Pós-Natal de Edimburgo (EPDS), considerado bom método de triagem,^13^ que compreende um questionário com dez perguntas, pontuadas numa escala de risco, que classifica a doença desde a forma leve àquela de alta gravidade.

No que diz respeito ao tratamento da depressão, o uso de antidepressivos melhora os sintomas da doença,^18^ favorece a aderência às demais terapêuticas e demonstra tendência na redução de eventos cardiovasculares maiores, no entanto, sem evidências na redução da mortalidade geral ou cardiovascular. Os melhores resultados do tratamento são potencializados com a psicoterapia, especialmente a terapia cognitivo-comportamental, e a prática de exercícios físicos ( Tabela 1 ).

Os inibidores seletivos de recaptação da serotonina (ISRS) são os antidepressivos considerados como de primeira linha, inclusive no tratamento da depressão puerperal, destacando-se o uso da sertralina, nas mulheres lactantes, pela baixa concentração no leite materno e boa eficácia no tratamento não inferior ao tempo de 6 a 12 meses após o diagnóstico. Nos casos moderados a graves, tem sido indicada a brexanolona, recentemente aprovada pela *Food and Drug Administration* (FDA) como fármaco bem tolerado, eficaz e específico para depressão puerperal.^18^

Quanto aos efeitos do uso prolongado de antidepressivos, um estudo de coorte recente demonstrou que a utilização de ISRS ao longo de dez anos promoveu a redução de 32% do risco de diabetes e de 23% de hipertensão arterial.^19^ O estudo também evidenciou aumento do risco de doença coronariana e de mortalidade geral e cardiovascular, particularmente associados ao uso de outros antidepressivos (mirtazapina, venlafaxina, duloxetina, trazodona),^18^ chamando a atenção para o fato de que algumas classes de antidepressivos podem oferecer melhores benefícios que outras, no cuidado à saúde cardiovascular.

É oportuno mencionar que a serotonina, conhecida como 5-hidroxitriptamina (5-HT), além de neurotransmissor do sistema nervoso central, também desempenha um papel significativo nos tecidos periféricos. Há crescentes indícios sugerindo que a serotonina interfere nas respostas das células imunes e, contribui para o desenvolvimento da doença cardiovascular e outras doenças consequentes da hiperatividade do sistema imunológico. Admite-se que a serotonina deflagra processos inflamatórios em condições fisiológicas normais e intensifica o estado pró-inflamatório em condições patológicas que envolvem inflamação, como a aterosclerose.^20^

Por último, é importante reforçar que a busca de convicções psicológicas positivas, tais como otimismo, senso de propósito, afetos positivos, sentimentos de gratidão e resiliência, e não apenas o controle dos fatores negativos, determinam a redução dos eventos agudos e o melhor controle dos fatores de riscos da DCV em mulheres que sofrem de depressão ( Tabela 1 ).^21 , 22^

## Conclusões

As crescentes evidências estabelecem a depressão como um fator de risco modificável para a DCV, e diante do reconhecimento da sua alta prevalência entre as mulheres, é imprescindível que o aprimoramento da investigação e o implemento de estratégias efetivas de abordagem das distintas formas de depressão devam ser reforçadas em diretrizes de prevenção da DCV na mulher.
