# Supplementary material for: Depressão, Doença Cardiovascular e Sexo Feminino: Uma Tríade Subestimada
Source: Arq Bras Cardiol. 2023 Jun 23;120(7):e20220858. [Article in Portuguese] doi: 10.36660/abc.20220858 (PMC10364992; doi:10.36660/abc.20220858)
Supplement: Translation

# Translation: Depression, Cardiovascular Disease, and Female Gender: An Underestimated Triad

**Authors:** Walkiria Samuel Avila, Maria Alayde Mendonça Rivera, Ivan Romero Rivera

**Affiliations:** 1 Brazil Instituto do Coração do Hospital das Clínicas da Faculdade de Medicina da Universidade de São Paulo, São Paulo, SP – Brazil; 2 Brazil Universidade Federal de Alagoas – Faculdade de Medicina, Maceió, AL – Brazil

**Keywords:** Depression, Cardiovascular Diseases, Female Gender, Immune System, Hypothalamo-Hypophyseal System, Renin Angiotrensin Aldosterone System

## Introduction

Depression is a global disease affecting around 330 million people, corresponding to 4.4% of the global population, and is the second cause of incapacitation in the USA.^1^In Brazil, it is estimated that 5.8% of the population, 11 million people, suffer from this disease, placing the country first in Latin America and the second in the Americas considering the number of cases.^1^In addition, the data published by the National Health Survey (PNS) conducted in 2013 verified that 78.8% of Brazilian people with significant depressive symptoms were not receiving any treatment.^2^

### Depression, women, and cardiovascular risk

Women, since their teenage years, are twice as likely to develop depression compared to men; the early the disease appears, the more severe the prognosis. It is estimated that around 20 to 25% of women will suffer at least one depression episode throughout their lives, with a high rate of disease recurrence, which, in its turn, is related to an increased risk of cardiovascular disease (CVD).^3^

Within this scenario, the contemporary world highlights CVD as the main reason for death in both sexes, representing 56% of the mortality among women.^4^It is worth emphasizing that depression is frequently identified among CVD patients, besides evidence that depression and CVD present a bidirectional relationship, which means that they are predictive “among themselves,” and combined, they set off more severe cardiac events ( Figure 1 ).^5 , 6^

**Figure 1:** – Pathophysiology of depression and cardiovascular disease.

There is evidence that depression and CVD share a pathogenesis related to the immune system, with the hypothesis that the persistence of a chronic inflammatory state, with increased expression of inflammatory cytokines, acute-phase proteins, and adhesion molecules, when associated with the deregulation of the hypothalamic-pituitary-adrenal axis, of the renin-angiotensin-aldosterone system and the serotonin-kynurenine^7^pathways contribute to the development of both diseases ( Figure 1 ).^7^

The reasons for the prevalence in the female gender are not yet clear; however, it is assumed that, beyond the genetical predisposition, biological, psychosocial, and family factors, there is a connection between depression and hormonal fluctuations, modulated by the levels of estrogen and progesterone, especially those inherent to the pregnancy and childbirth cycle and the menopause ( Figure1).^8^

### Puerperal depression

In this context, it is worth highlighting the importance of post-partum depression, with a prevalence of around 20.5% in Brazil, which may be underestimated due to the lack of diagnosis and omission in notifications.^9^A recent study should be mentioned, which demonstrated a higher proportion of myocardial infarction and cerebrovascular accidents among women who suffered post-partum depression, a theme that must be further studied to track a new predictor of cardiovascular risk in women.^10^

The clinical condition of post-partum depression is no different from the classic conditions for the general population ( Table 1 ), and it can begin during pregnancy or up to four weeks after childbirth.^11^Among the symptoms, we highlight mood changes, loss of interest in life, changes in appetite and sleep, incapacity to focus, low self-esteem, and suicide/homicide thoughts. This scenario can progress to psychosis (agitation, confusion, delusions) and culminate with suicide.

**Table 1:** – Characteristics of female gender depression and puerperal depression

	Depression	Post-partum depression
Risk Factors	Family history of depression Chronic diseases (multiple sclerosis, diabetes mellitus, cancer), Chronic pain, sleep changes Hormonal fluctuations (premenstrual, pregnancy, perimenopause) Changes in the circadian rhythm Nutritional deficiency Stress, Loss, Mourning Medicines (anticonvulsants, benzodiazepine, corticosteroids) Abuse of alcohol and other drugs	Depression during pregnancy Multiparous women (≥ 3 births) Severe stress during pregnancy Current or previous sexual abuse Marital or partner dissatisfaction Complications referring to the birth or the newborns Mixed feelings about the pregnancy (planned or not) Lack of emotional support by the spouse, partner, family/friends **PROTECTIVE FACTORS** Higher maternal and paternal schooling Presence of spouse or partner Support from the child’s father and family during pregnancy
Observed Symptoms	Depressed mood; Lack of interest or pleasure in usual activities; Insomnia or hypersomnia; Significant weight gain or loss (> 5%/month), reduction or increase in appetite Psychomotor retardation or agitation Fatigue and low self-esteem; Reduction in the ability to focus or make decisions Thoughts of uselessness or excessive or inadequate guilt Recurring death or suicide thoughts, or attempt at suicide	Changes in appetite and eating habits Extreme fatigue, Insomnia Mood changes (irritability, anger, and sadness) Lack of interest in pleasant or favorite activities Low self-esteem or ability to focus Social isolation, including the baby, associated with feelings of guilt/shame Feelings of sadness, unrest, anxiety, or hopelessness Persistent complaints (headache, stomachache, or others) Thoughts about hurting herself or the baby (suicidal/homicidal thoughts)
Diagnostic Tool	2-Question Patient Health Questionnaire (PHQ-2) 9-Question Patient Health Questionnaire (PHQ-9)	Edinburgh Postnatal Depression Scale (EPDS)
Treatment	Psychotherapy (cognitive behavioral therapy; interpersonal therapy; support groups); Physical exercises Antidepressants: selective serotonin reuptake inhibitors; serotonin/norepinephrine reuptake inhibitors (fluoxetine and paroxetine inhibit the effect of tamoxifen and should not be used in patients with breast cancer); selective norepinephrine/dopamine reuptake inhibitors; alpha-1 receptor antagonists; serotonin modulators. Tricyclic antidepressants must be avoided in patients with structural cardiovascular disease due to their effects on the conduction of cardiac stimuli. Electroconvulsive therapy (severe depression) Orientation for searching for positive psychological factors	Psychotherapy (cognitive behavioral therapy, interpersonal therapy, support groups). Antidepressants: selective serotonin reuptake inhibitors in the initial treatment (sertraline is the preferred drug due to its low concentration in mother’s milk); Other indicated drugs: paroxetine, duloxetine, nortriptyline, and imipramine; Tricyclic antidepressants can be considered the first-choice treatment in case of a history of previous successful treatment and provided there are no counter-indications to its use, such as the probability of suicide; doxepin is counter-indicated (respiratory depression; sedation; hypotonia) Brexanolone IV in severe cases (available in the USA) Physical exercise Other therapies (yoga; massages; relaxation techniques; meditation) Electroconvulsive therapy (severe depression, with hallucinations, illusions, and suicidal thoughts) Lactation must be encouraged due to its benefits for the mother and child relationship; the risks of the treatment for the baby must be discussed, as well as the risks of not treating the depression for the family dynamics.
Prognosis	Failure to recognize/treat the depression can cause: a) negative impact in recognizing and controlling cardiovascular risk factors b) increased incidence of acute events	Failure to recognize/treat post-partum depression can cause: a) increased the risk of suicide and infanticide; b) rupture of the mother-child bond, with physical/emotional damages with negative impacts on the child’s growth and development (prone to obesity; difficulty in social interactions)

Post-partum depression harms the quality of life, weakens the mother-child bond, favors marital discord, and motivates infanticide, besides mother suicide. In this aspect, the most alarming fact is that post-partum depression is a psychiatric complication that increases by five times suicide attempts, and it was included as the cause among 30 to 50% of pregnant women who committed suicide.^12^Given the reasons presented, the foresight of medical professionals in diagnosing depression in women is extremely important, once 10 to 20% of patients deny the disease and omit symptoms.^13^

### Perimenopause depression

Still, in the women’s biological cycle, perimenopause is a vulnerable window for the development of depression, even among women who have no history of the disease. Among risk factors for depression in perimenopause, we highlight anxiety and previous depressive disorders, as well as other conditions, such as black race, obesity, tragic events throughout life, and social isolation.^14^The depression “triggers” in perimenopause lie in career or relationship changes, awareness of aging, the transformation of physical features, and personal or family diseases, besides the known “empty nest” syndrome.

The usual depression symptoms are overlapped with the ones specific to menopause, such as heat waves, night sweats, sleep and libido disorders, and cognitive changes, making it harder for the disease to be diagnosed.^13^In this sense, the application of conventional questionnaires (Menopause Rating Scale – MRS, Menopause Quality of Life Questionnaire – MENQOL, Greene Climacteric Scale, Utian Quality-of-Life Scale) can contribute to the interface of symptoms (menopause, anxiety, depression), and guide towards a safe and efficient approach to the disease.^15^

### Depression, pandemic COVID-19, and gender female

A comprehensive review of the interaction between gender, depression, CVD, and COVID-19 highlighted a combined 25% prevalence (95% CI: 18%-33%) of depression in the general population resulting from the effects of the COVID-19 pandemic. Interestingly, women seem to have less severe complications in the short term when diagnosed than men but suffer from greater complications from COVID-19 over time, including depression and worsening lifestyle habits, increasing cardiovascular risk.^16^

The female gender has been highlighted as a significant risk factor for worsening health status and symptoms of depression, probably due to the higher prevalence of depressive disorders and pre-existing anxiety, constant environmental stress, and domestic violence, which have intensified during the COVID-19 pandemic. Furthermore, women experience stressful situations related to the reproductive period, such as fertility problems, pregnancy, miscarriage, post-partum depression, and intimate partner violence.

A multicenter study that assessed the mental health status of pregnant and breastfeeding women during the COVID-19 pandemic showed a prevalence of 15% of major depressive symptoms among 3,907 pregnant women and 13% among 5,134 breastfeeding mothers. This study reinforced the importance of monitoring the perinatal mental condition during pandemics and other social crises to safeguard maternal and child health.^17^

### Depression, diagnosis, and treatment in women

The usual recommendation to diagnose depression is the application of the Patient Health Questionnaire (PHQ)-2 method, which includes two questions and, given the suspicion of the disease, the Patient Health Questionnaire (PHQ)-9, which contains nine questions, to confirm the diagnosis. The use of both depression investigation methods in clinical practice has shown good sensitivity, specificity, and negative predictive value. They are available at https://bit.ly/2VvPHIG (PHQ-2) and https://bit.ly/2PY3INz (PHQ-9).

In the investigation of post-partum depression, it is recommended to apply the Edinburgh Postnatal Depression Scale (EPDS), considered a good triage method,^13^which is composed of a questionnaire with ten questions, scored in a risk scale, which classified the disease from light to high severity.

Regarding the treatment of depression, the use of antidepressants improves the symptoms of the disease,^18^favors adherence to other therapies, and shows a tendency to reduce bigger cardiovascular events; however, with no evidence of the reduction of general or cardiovascular mortality. The best results of the treatment are potentialized with psychotherapy, especially cognitive behavioral therapy, and the practice of physical exercise ( Table 1 ).

Selective serotonin reuptake inhibitors (SSRIs) are considered the first-line antidepressants, including in the treatment of post-partum depression, with a highlight on the use of sertraline, in lactating women, due to its low concentration in mother milk and good efficiency in the treatment not shorter than 6 to 12 months after the diagnosis. In moderate to severe cases, brexanolone is being indicated, which was recently approved by the Food and Drug Administration (FDA) as a well-tolerated, efficient, and specific drug for post-partum depression.^18^

Regarding the effects of the long-term use of antidepressants, a recent cohort study showed that using SSRIs for ten years was associated with a 32% reduction in the risk of diabetes and a 23% reduction in arterial hypertension.^19^The study also showed an increase in the risk of coronary disease and general/cardiovascular mortality, particularly associated with the use of other antidepressants (mirtazapine, venlafaxine, duloxetine, trazodone),^18^reinforcing that some classes of antidepressants may offer better benefits than others in health care cardiovascular.

It is opportune to comment that serotonin, also known as 5-hydroxytryptamine (5-HT), is a neurotransmitter in the central nervous system and plays a significant role in peripheral tissues. Growing evidence suggests that serotonin interferes with immune cell responses and contributes to the development of cardiovascular disease and other diseases resulting from an immune hyperactivity system. It is assumed that serotonin triggers inflammatory processes under normal physiological conditions and intensifies the pro-inflammatory state in pathological conditions involving inflammation, such as atherosclerosis.^20^

Finally, it is very important to reinforce that the search for positive psychological convictions, such as optimism, a sense of purpose, positive affections, feelings of gratitude, and resilience, and not only the control of negative factors, determines that reduction of acute events and the better control of CVD risk factors in depression patients ( Table 1 ).^21 , 22^

## Conclusions

The growing evidence establishes depression as a modifiable risk factor for CVD. Given the recognition of its high prevalence among women, it is fundamental that the improvement of the investigation and implementation of effective strategies to approach the different types of depression must be reinforced in guidelines for CVD prevention in women.
